# Efficacy of Electrochemotherapy with Bleomycin, Oxaliplatin, or Oxaliplatin with Bevacizumab in the Treatment of Colorectal Hepatic Metastases in Rats

**DOI:** 10.3390/cancers17172753

**Published:** 2025-08-23

**Authors:** Antonios E. Spiliotis, Orestis Mallis Kyriakides, Sebastian Holländer, Gudrun Wagenpfeil, Matthias W. Laschke, Matthias Glanemann, Gereon Gäbelein

**Affiliations:** 1 Department of Surgery, Campus Charité Mitte, Campus Virchow Klinikum, Charité Universitätsmedizin Berlin, 13353 Berlin, Germany; 2 Institute for Clinical and Experimental Surgery, Saarland University, PharmaScienceHub (PSH), 66421 Homburg, Germany; matthias.laschke@uks.eu; 3 Department of Cardiovascular Surgery, Heart Center Niederrhein, Helios Hospital Krefeld, 47805 Krefeld, Germany; orestis.malliskyriakides@helios-gesundheit.de; 4 Department of General Surgery, Vascular-, Visceral- and Pediatric Surgery, Saarland University Medical Center, 66421 Homburg, Germany; sebastian.hollaender@uks.eu (S.H.); matthias.glanemann@uks.eu (M.G.); gereon.gaebelein@uks.eu (G.G.); 5 Institute for Medical Biometry, Epidemiology and Medical Informatics, Saarland University Medical Center, 66421 Homburg, Germany; gudrun.wagenpfeil@uni-saarland.de

**Keywords:** Electrochemotherapy, bleomycin, oxaliplatin, bevacizumab, colorectal cancer

## Abstract

Background/Objectives: Electrochemotherapy (ECT) has been shown to be effective in treating colorectal liver metastases when combined with bleomycin (BLM). Based on this promising finding, we compared in this study the efficacy of BLM with oxaliplatin (OXP) and bevacizumab (BVZ) in ECT. Methods: WAG/Rij rats were randomized into three groups and underwent ECT with intravenous injection of BLM, OXP, or OXP with BVZ for eight days following hepatic tumor cell implantation. Ultrasound and photoacoustic imaging served to assess oxygen saturation (SO_2_) and hemoglobin concentration (HbT) of the developing tumors. Tissue samples were analyzed by histology and immunohistochemistry. Results: BLM treatment significantly reduced SO_2_ (33.7%) and HbT (12.7%) levels compared to pretreatment values. In contrast, the OXP-treated groups exhibited only modest reductions in both parameters. BLM also induced a markedly higher necrosis rate (82.6%) compared to OXP and OXP/BVZ (11.0% and 26.3%). Conversely, OXP-treated tumors exhibited higher apoptosis rates. Furthermore, BLM treatment led to a decrease in tumor cell proliferation and a reduction in inflammatory response compared to the other treatments. Notably, BLM caused a 26.2% reduction in CD31-positive microvessels, which was significantly higher than that observed in the OXP group. **Conclusions**: BLM showed a more effective anti-tumor activity than OXP, suggesting its preferred use as chemotherapeutic agent in ECT.

## Simple Summary

Electrochemotherapy (ECT) has been established as a treatment option for cutaneous and subcutaneous neoplastic lesions with response rates of up to 86%. These beneficial outcomes have increased the interest in utilizing this method for hepatic tumors as well. As a non-thermal technique, ECT may particularly offer advantages in high-risk anatomical regions by preserving the integrity of blood vessels and bile ducts. However, current evidence on the role of ECT in the treatment of hepatic metastases is limited. We compared in the present study the efficacy of bleomycin with other chemotherapeutic agents that are commonly used in the clinical treatment of hepatic metastases, i.e., oxaliplatin and bevacizumab. Our study highlights the superior efficacy of bleomycin compared to oxaliplatin in the treatment of colorectal hepatic metastases with ECT. While oxaliplatin remains a standard oncological treatment, it was less effective than bleomycin, even with bevacizumab addition.

## 1. Introduction

Electrochemotherapy (ECT), a non-thermal ablative procedure, combines chemotherapy with well-dosed electric pulses for reversible electroporation (rEP) of the cell membrane. Through rEP, a transmembrane voltage is induced on the cellular membrane that exceeds a certain threshold value, causing transient formation of nanoscale hydrophilic nanopores and increased cellular permeability [1,2]. This enhanced permeability facilitates the intracellular uptake of chemotherapeutic agents that would otherwise exhibit limited penetration into tumor cells, thereby augmenting their cytotoxic efficacy and promoting tumor cell death [2,3,4].

ECT is an established therapeutic modality for cutaneous and subcutaneous neoplastic lesions, demonstrating response rates of up to 86% [3,4,5]. The favorable outcomes achieved in these settings have prompted growing interest in extending its application to hepatic tumors. Nevertheless, the evidence supporting ECT for hepatic metastases remains scarce. To date, only small-scale clinical studies have evaluated the efficacy of ECT with bleomycin (BLM) in hepatic malignancies [6,7,8,9], and the potential utility of alternative chemotherapeutic agents has not been systematically explored.

In our previous studies, we demonstrated in a rat model that the combination of ECT with intravenous (i.v.) administration of BLM significantly increases necrosis of colorectal liver metastases (CRLM) by up to 87%, highlighting the potential of this modality for treating hepatic tumors [10,11]. Based on these promising findings, we compared in the present study the efficacy of BLM with other chemotherapeutic agents that are commonly used in the clinical treatment of CRLM, i.e., oxaliplatin (OXP) and bevacizumab (BVZ).

## 2. Materials and Methods

### 2.1. Animals

Twenty-four WAG/Rij rats (males: n = 12, females: n = 12) with a body weight of 196.0 ± 2.6 g and an age of 46.0 ± 1.9 weeks were used for the experiments (Institute for Clinical and Experimental Surgery, Saarland University, PharmaScienceHub (PSH), Homburg, Germany). Animals were housed in groups under temperature- and humidity-controlled conditions with a 12 h light/dark cycle and had ad libitum access to water and standard laboratory chow (Altromin, Lage, Germany). 

All procedures were carried out in accordance with the European Directive 2010/63/EU on the protection of animals used for scientific purposes and the National Institutes of Health Guide for the Care and Use of Laboratory Animals [12]. The study protocol was approved by the State Office for Consumer Protection, Saarbrücken, Germany (permission no. 21/2019).

### 2.2. Experimental Protocol

The rats were randomized into three groups, each comprising four males and four females. On day 0, all animals underwent laparotomy with tumor cell injection into the left liver lobe. On day 8, relaparotomy was performed, and tumor development was assessed by ultrasound and photoacoustic imaging. Thereafter, the animals were treated as follows: (i) ECT with i.v. administration of BLM (BLM group), (ii) ECT with i.v. injection of OXP (OXP group), or (iii) ECT with combined i.v. administration of OXP and BVZ (OXP/BVZ group). Chemotherapy was administrated in the inferior vena cava, because in our previous study it has been proven that i.v. application is associated with better oncological outcomes compared to direct intratumoral injection [11].

On day 13, corresponding to the fifth day after treatment, animals underwent relaparotomy for final tumor assessment by ultrasound and photoacoustic imaging, and for collection of venous blood samples. Subsequently, animals were euthanized by i.v. overdose of sodium pentobarbital. The left liver lobe, including both tumor and adjacent normal hepatic tissue, was then harvested for histological and immunohistochemical analyses.

Throughout the experiments, the animals received daily analgesic treatment with tramadol (40 mg/mL in drinking water). On the days of procedures, they additionally received carprofen (5 mg/kg body weight, subcutaneously) and buprenorphine (0.05–0.1 mg/kg body weight, subcutaneously) intraoperatively.

### 2.3. Tumor Cell Injection

CC531 rat colon carcinoma cells (CLS, Heidelberg, Germany), which are syngeneic to WAG/Rij rats, were cultured as described previously [13]. Under isoflurane anesthesia, we conducted a median laparotomy with subcapsular injection of 5 × 10^5^ CC531 cells (in 50 µL phosphate-buffered saline) on the lower surface of the left liver lobe [10].

### 2.4. ECT

The ECT procedure has been previously described in detail [10,11]. Briefly, BLM (BLEO-cell^®^ 15 mg, STADAPHARM GmbH, Bad Vilbel, Germany) was administered through i.v. at a concentration of 4 U/kg body weight [14] and OXP (Oxaliplatin STADA^®^ 5 mg/mL, STADAPHARM GmbH, Bad Vilbel, Germany) at a concentration of 5 mg/kg body weight [15]. In the OXP/BVZ group, OXP injection was followed by an i.v. injection of BVZ (Oyavas^®^ 25 mg/mL, STADAPHARM GmbH, Bad Vilbel, Germany) at a concentration of 5 mg/kg body weight [16,17]. ECT was performed using the Sennex^®^ Tumor System (BIONMED^®^ Technologies GmbH, Saarbrücken, Germany). Eight electric pulses, each with a duration of 100 μs, were delivered between the two electrodes three minutes after chemotherapy administration [18,19]. In the OXP/BVZ group, rEP was conducted three minutes after i.v. administration of OXP. In accordance with the European guidelines for ECT application, the Sennex^®^ Tumor System delivered a voltage of 1000 V between the electrodes, corresponding to an amplitude of 125 V/mm and a frequency of 1 Hz [5,20].

### 2.5. Ultrasound and Photoacoustic Imaging

Ultrasound and photoacoustic imaging of the newly developing tumors was conducted using the Vevo LAZR system 2100 (FUJIFILM VisualSonics Inc., Toronto, ON, Canada) in conjunction with a real-time microvisualization LZ550 linear-array transducer (FUJIFILM VisualSonics Inc.), which has a center frequency of 40 MHz. The ultrasound examination was performed during laparotomy on day 8 prior to treatment and on day 13 before euthanizing the animals. The procedure adhered to established standards [10].

Photoacoustic imaging in OxyHemo mode was acquired at two wavelengths, 750 nm and 850 nm, utilizing a two-dimensional photoacoustic gain of 42 dB and a hemoglobin threshold of 20 dB. These wavelengths were selected to facilitate the determination of oxygen saturation (SO_2_) by differentiating signals from oxygenated and deoxygenated hemoglobin [21]. Additionally, at these wavelengths, potential artifacts due to edema resulting from ECT were minimized, as water absorption in this range is relatively low [22]. Hemoglobin concentration (HbT) and SO_2_ were quantified across the entire tumor tissue.

### 2.6. Analysis of Blood Samples

Venous blood samples were collected via puncture of the subhepatic vena cava on day 13 immediately following ultrasound imaging. The total leukocyte count (10^9^/L), lymphocyte count (10^9^/L), monocyte count (10^9^/L), neutrophil count (10^9^/L), erythrocyte count (10^12^/L), and platelet count (10^9^/L), along with hemoglobin concentration (g/dL) and hematocrit (%), were analyzed using a cell counter (VetScan HM5; Scil Animal Care Company GmbH, Viernheim, Germany). Liver function parameters were not assessed, as our previous study demonstrated that ECT does not impair hepatic function [11].

### 2.7. Histological and Immunohistochemical Analysis

The histological and immunohistochemical analysis was performed according to our previous studies [10,11]. The tumor tissue and the surrounding normal hepatic parenchyma were fixed in 4% phosphate-buffered formalin, embedded in paraffin, and sectioned at a thickness of 3 μm. Hematoxylin and eosin staining was performed to assess tumor necrosis.

For immunohistochemical analyses, sections were stained with specific primary antibodies. Apoptotic cells were identified using a rabbit polyclonal anti-cleaved caspase-3 antibody (1:100; Cell Signaling Technology, Frankfurt, Germany). Proliferating cells were detected with a monoclonal mouse-anti-human anti-proliferating cell nuclear antigen (PCNA) antibody (1:100; Dako, Hamburg, Germany). Microvessels were visualized using a polyclonal rabbit anti-CD31 antibody (1:200; Abcam, Cambridge, UK), and neutrophilic granulocytes were labeled with a rabbit polyclonal anti-myeloperoxidase (MPO) antibody (1:100; Abcam, Cambridge, UK). The appropriate secondary antibodies were applied according to standard immunohistochemical protocols as reported in detail in our previous studies [10,11].

For histological and immunohistochemical analyses, a BX60 microscope (Olympus, Hamburg, Germany) in conjunction with the imaging software cellSens Dimension 1.15 (Olympus, Hamburg, Germany) was employed. To ensure objectivity, the sections were coded and independently analyzed by three researchers who were blinded to the treatment groups.

The extent of necrosis within the tumor tissue was quantified as a percentage of the total tumor area in the section displaying the largest cross-sectional tumor diameter. Cell proliferation was assessed using a semiquantitative index based on the proportion of PCNA-positive tumor cells relative to the total tumor cell count in 10 high-power fields (HPF) of non-necrotic tumor tissue. The specimens were classified into one of five categories: 0 (<1%), 1 (1–10%), 2 (11–30%), 3 (31–50%), and 4 (>50%) PCNA-positive cells.

For the quantification of apoptotic cells, inflammatory cells, and microvessels, counts were performed in 10 randomly selected HPF within non-necrotic tumor regions, with five fields analyzed in the tumor center and five fields in the periphery. The number of positive cells was expressed as an absolute count per HPF, while microvessel density was recorded as the number of CD31-positive microvessels per mm^−2^.

### 2.8. Statistical Analysis

As the sample sizes in all groups were fewer than 12, parametric tests were applied in all cases. All values are presented as mean ± SEM (standard error of the mean). Group differences were analyzed using a one-way analysis of variance (ANOVA). Given the exploratory nature of the study, adjustments for multiple post hoc testing were not applied. Pairwise comparisons were conducted using Student’s *t*-test. Statistical significance was determined based on a two-sided significance level of 0.05. All statistical analyses were performed using IBM SPSS, version 28.0.1.0.

## 3. Results

### 3.1. Tumor Development and General Health Conditions

All animals developed a hepatic tumor in the left liver lobe by day 8 following tumor cell injection, with no evidence of peritoneal or extrahepatic metastases. Regular assessments of body weight indicated a slight, non-significant decline from day 0 to day 13, not exceeding 10% of the total body weight. Throughout the observation period, the animals exhibited no systemic effects from tumor progression and maintained normal activity, feeding behavior, and grooming habits. No complications related to ECT were observed in this study.

### 3.2. Ultrasound and Photoacoustic Imaging

**Figure 1:**
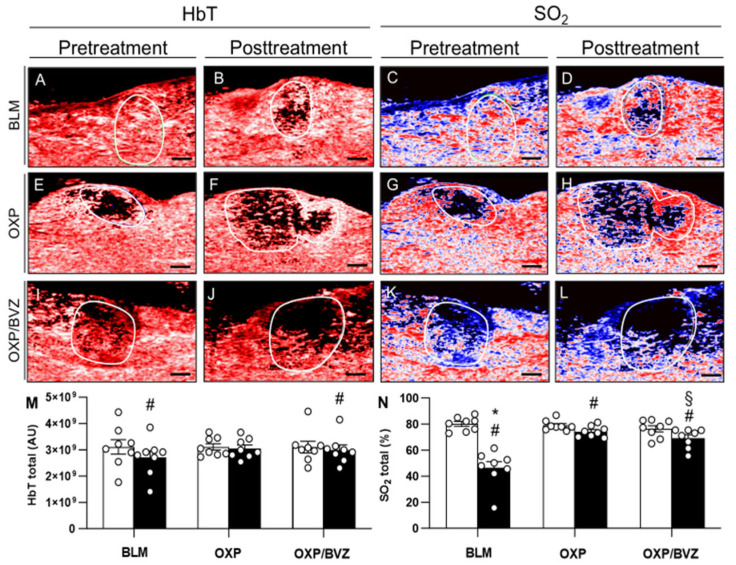
(**A**–**L**) Photoacoustic imaging with pretreatment (on day 8) and posttreatment (on day 13) hemoglobin map (HbT) and oxygen saturation map (SO_2_) in animals treated with ECT and BLM (**A**–**D**), OXP (**E**–**H**), or OXP/BVZ (**I**–**L**). In the oxygen saturation map, the lowest saturation is dark blue, whereas the highest levels are red. White lines indicate the tumor outline. Scale bars: 1 mm. (**M**,**N**) HbT and SO_2_ were measured in the whole tumor area. Data are given as mean ± SEM; ^#^ *p* < 0.05 vs. pretreatment levels; * *p* < 0.05 vs. SO_2_ posttreatment in OXP and OXP/BVZ; ^§^ *p* < 0.05 vs. SO_2_ posttreatment in OXP.

On day 8 before treatment, ultrasound and photoacoustic analyses were conducted to evaluate the pretreatment imaging characteristics of the tumors. At this time point, tumor volumes (BLM: 33.0 ± 3.6 mm^3^; OXP: 30.5 ± 2.5 mm^3^; OXP/BVZ: 35.8 ± 3.2 mm^3^), HbT, and SO_2_ were comparable among the three groups (Figure 1).

HbT and SO_2_ were additionally assessed five days following the ECT procedure (day 13), whereas the tumor volume could not be exactly measured anymore due to edema formation. Regarding HbT, the BLM group exhibited the most substantial decrease within the treated tissue, with a reduction of approximately 12.7%. The OXP and OXP/BVZ groups also showed declines, though to a lesser extent (2.1% and 3.4%, respectively). Compared to pretreatment values, SO_2_ levels showed the most extensive reduction in the BLM group (33.7%), whereas the decreases in the OXP and OXP/BVZ groups were less pronounced (4.7% and 12.1%, respectively) (Figure 1).

### 3.3. Tumor Necrosis

Histological examination of hematoxylin-eosin-stained sections revealed treatment-induced tumor necrosis, encased by a fibrotic pseudocapsule composed of fibroblasts, infiltrating inflammatory cells, and proliferating microvessels exhibiting red blood cell (RBC) extravasation. In the periphery of the treated area, a limited number of residual tumor cells were detected, accompanied by inflammatory cell infiltration. The fibrotic pseudocapsule was bordered by regenerative marginal hepatocytes and normal hepatic parenchyma, with no evidence of tumor infiltration. Notably, the walls of blood vessels and bile ducts adjacent to the ablation zone remained intact in all groups.

**Figure 2:**
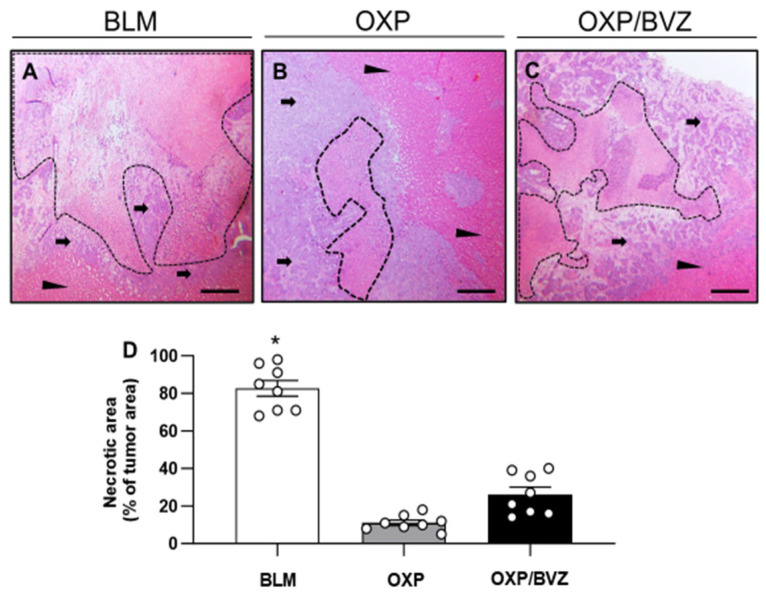
(**A**–**C**) Histological analysis of necrotic tumor cell death (borders marked by dotted line) in animals treated with ECT and BLM (**A**), OXP (**B**), or OXP/BVZ (**C**). The fibrotic pseudocapsule with residual tumor cells is marked by arrows and normal hepatic tissue by arrowheads. Scale bars: 50 µm. (**D**) Necrotic areas in the tumor tissue were measured as percentage of the whole tumor area. Data are given as mean ± SEM; * *p* < 0.05 vs. OXP and OXP/BVZ.

In the histological analysis, the BLM group exhibited the highest proportion of necrotic tissue, accounting for 82.6 ± 0.1% of the treated area. In contrast, the rate of necrotic tissue was significantly lower in the OXP (11.0 ± 0.0%) and OXP/BVZ (26.3 ± 0.1%) groups (Figure 2).

### 3.4. Apoptotic Cell Death and Tumor Cell Proliferation

**Figure 3:**
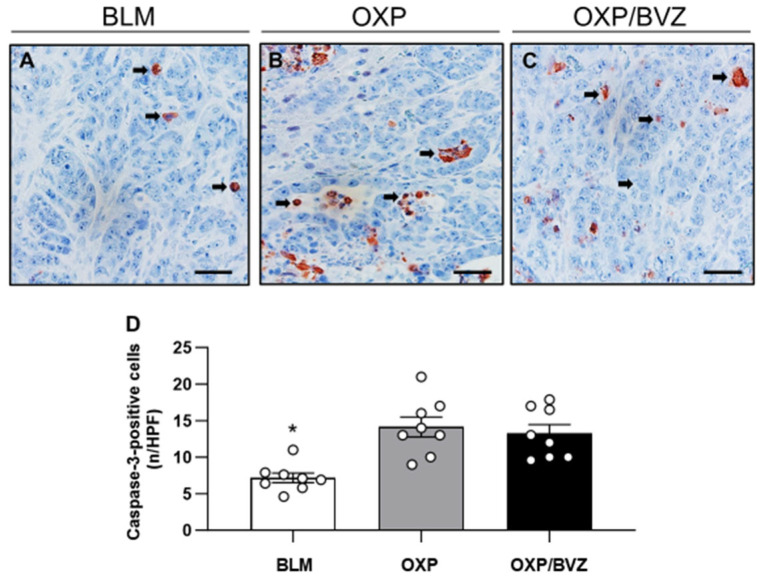
(**A**–**C**) Immunohistochemical analysis of cleaved caspase-3-positive tumor cells in animals treated with ECT and BLM (**A**), OXP (**B**), or OXP/BVZ (**C**). Apoptotic cells (arrows) are stained brown. Scale bars: 50 µm. (**D**) The diagram displays the mean number of cleaved caspase-3-positive cells in the tumor tissue per HPF. Data are given as mean ± SEM; * *p* < 0.05 vs. OXP and OXP/BVZ.

A limited number of cleaved caspase-3-positive apoptotic cells was detected at the periphery of the ablation zone, among residual tumor cells, and within the fibrotic pseudocapsule (Figure 3). Treatment with OXP and OXP/BVZ induced nearly a twofold increase in apoptotic cells compared to the BLM group (Figure 3).

**Figure 4:**
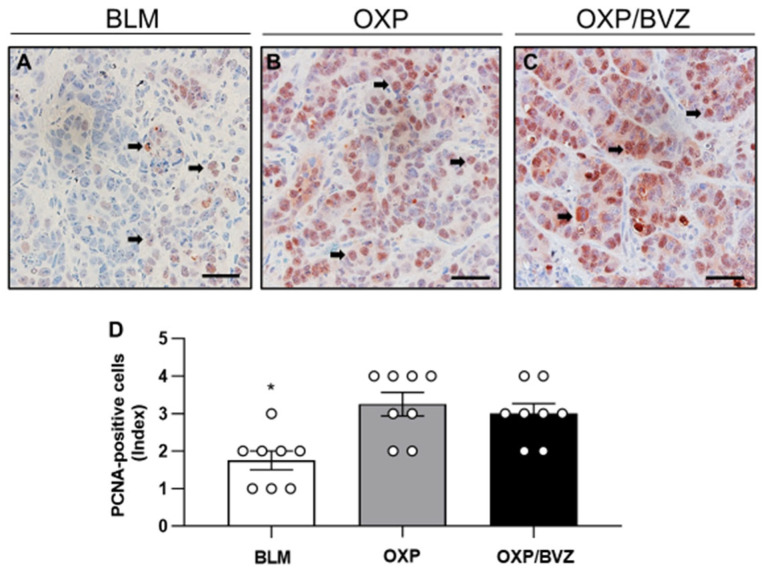
(**A**–**C**) Immunohistochemical sections of PCNA expression in the tumor tissue of animals undergoing ECT with BLM (**A**), OXP (**B**), or OXP/BVZ (**C**). PCNA-positive cells (arrows) are stained brown. Scale bars: 50 µm. (**D**) The diagram displays the quantitative analysis of PCNA-positive cells (semiquantitative index) in the tumor tissue per HPF. Data are given as mean ± SEM; * *p* < 0.05 vs. OXP and OXP/BVZ.

Furthermore, immunohistochemical assessment of tumor cell proliferation demonstrated a substantial proportion of PCNA-positive cells in the OXP and OXP/BVZ groups, comprising up to 50% of the total cell population (Figure 4). In contrast, BLM treatment significantly reduced tumor cell proliferation compared to the two other groups (Figure 4).

### 3.5. Tumor Vascularization and Inflammatory Response

**Figure 5:**
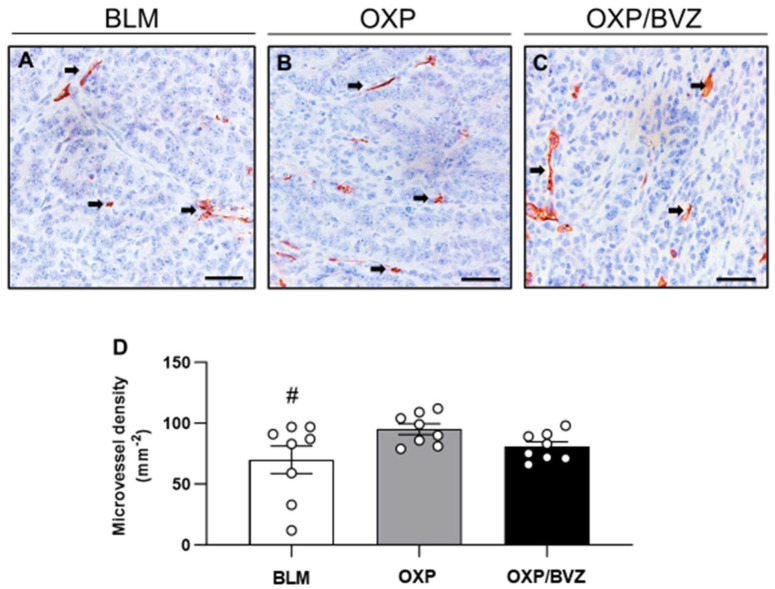
(**A**–**C**) Immunohistochemical analysis of CD31 expression in the tumor tissue of animals treated with ECT and BLM (**A**), OXP (**B**), or OXP/BVZ (**C**). CD31-positive blood vessels (arrows) are stained red. Scale bars: 50 µm. (**D**) The diagram shows the quantitative analysis of CD31-positive blood vessels in the tumor tissue. The microvessel density is given as the number of CD31-positive vessels (in 10 HPF) per mm2; ^#^ *p* < 0.05 vs. OXP.

The immunohistochemical analysis of CD31-positive microvessels revealed a reduction in microvessel density within the tumor tissue, with a 26.2% decrease in the BLM group and a 15.2% decrease in the OXP/BVZ group compared to the OXP group (Figure 5).

**Figure 6:**
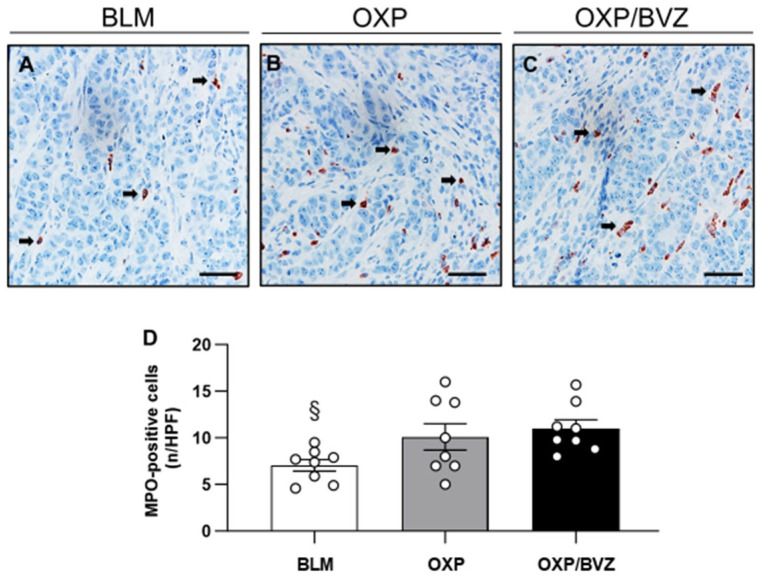
(**A**–**C**) Immunohistochemical analysis of MPO expression in the tumor tissue of animals treated with ECT and BLM (**A**), OXP (**B**), or OXP/BVZ (**C**). MPO-positive cells are stained red (arrows). Scale bars: 50 µm. (**D**) The diagram displays the quantitative analysis of MPO-positive cells in the tumor tissue per HPF. Data are given as mean ± SEM; ^§^ *p* < 0.05 vs. OXP/BVZ.

Similarly, the number of MPO-positive neutrophils was lower in the BLM-treated group than in the OXP and OXP/BVZ groups. Inflammatory cell infiltration was significantly higher in the OXP/BVZ group compared to the BLM group (Figure 6).

### 3.6. Blood Sample Analysis

The hematological analyses across all treatment groups revealed that white blood cell counts, including leukocytes, lymphocytes, and monocytes, remained within standard reference ranges. Specifically, leukocyte, lymphocyte, and monocyte counts were slightly elevated in the BLM and OXP groups compared to the OXP/BVZ group; however, all measurements remained within normal limits. Additionally, RBC, platelet, hemoglobin, and hematocrit levels were stable and within physiological ranges, though the OXP/BVZ group exhibited marginally higher hemoglobin and hematocrit levels. These variations did not reach statistical significance (Table 1).

## 4. Discussion

The demonstrated efficacy of ECT in the treatment of cutaneous cancer has led to growing interest in its application for hepatic tumors. As a non-thermal technique, ECT may particularly offer advantages in high-risk anatomical regions by preserving the integrity of blood vessels and bile ducts [23,24]. Previous studies have shown that ECT with i.v. administration of BLM induces extensive tumor necrosis, reduces vascularization, and decreases oxygenation within the treated tissue [10,11]. However, the efficacy of commonly used chemotherapeutic agents for CRLM in combination with ECT remains unknown. Therefore, the present study evaluated the efficacy of BLM, OXP, and OXP in combination with BVZ during ECT in a rat liver metastasis model.

OXP is a cornerstone of systemic therapy for CRLM, most commonly administered within FOLFOX doublets or FOLFOXIRI triplets, and is recommended perioperatively for resectable or potentially resectable disease in selected patients. BVZ, an anti-VEGF antibody, is recommended in combination with fluoropyrimidine oxaliplatin or irinotecan doublets for first-line treatment of initially unresectable CRLM [25,26]. As BVZ is not used as monotherapy in clinical practice, we did not include a BVZ monotherapy group or its combination with BLM, as this regimen is not clinically applicable.

Initially, we confirmed that all included animals exhibited consistent tumor development and comparable tumor characteristics. Sonographic and photoacoustic assessments conducted on the day of treatment showed that tumor volume, tumor SO_2_, and HbT were similar across all study groups. Consequently, the different treatment approaches were evaluated in tumors with uniform biological characteristics as well as comparable volume and vascularization.

Necrosis is a critical factor in evaluating the efficacy of ECT treatment. In our study, BLM treatment resulted in significantly higher rates of necrosis compared to the other two groups. This outcome can be attributed to multiple factors, including ECT-induced alterations in tumor vascularization as well as the direct cytotoxic effects of ECT and BLM.

It has been demonstrated that the efficacy of ECT is enhanced through the ‘vascular disrupting effect’ and the ‘vascular lock effect’ [2,4]. According to the ‘vascular disrupting effect’, endothelial cells lining the blood vessels within tumors are exposed to an electric field approximately 40% stronger than that applied to the surrounding tumor cells during rEP [27]. This increased permeability facilitates the diffusion of chemotherapy into endothelial cells, leading to cellular damage, vascular occlusion, and subsequent ischemic death of tumor cells adjacent to the obstructed blood vessels [2,28]. BLM exhibits distinct vascular-disrupting properties, resulting in approximately 70% cessation of tumor blood flow [27,29,30].

On the other hand, the effect of OXP with rEP on the vascular endothelium has not been examined so far. Considering data from studies, where cisplatin has been utilized in combination with rEP, we know that platinum-based chemotherapy agents demonstrate less efficacy on vascular endothelial cells compared to BLM [31]. BLM directly causes double- and single-strand DNA breaks via free radical formation in the presence of oxygen and metal ions. This damage is rapid and lethal, especially in electroporated endothelial cells, which are highly permeable after rEP [27,32,33]. In contrast, OXP induces cell death primarily through DNA adduct formation and crosslinking, triggering DNA damage responses, cell cycle arrest, and apoptosis via both intrinsic and extrinsic pathways [34,35,36]. Actively dividing cells, particularly those in the G2/M phase of the cell cycle, are more susceptible to OXP-induced DNA damage. Endothelial cells are often in a quiescent or slow proliferating state, making them less susceptible to agents like OXP that target dividing cells [37].

With regard to the role of the ‘vascular lock effect’, it is well established that electrical stimulation of precapillary smooth muscle cells induces direct vasoconstriction, followed by an indirect, sympathetically mediated vasoconstriction of the afferent arterioles [2,4,28]. Moreover, exposure to electrical fields alters the morphology of vascular endothelial cells, leading to increased vascular resistance and modifications in endothelial cell-to-cell junctions [2]. Consequently, both the ‘vascular disrupting effect’ and the ‘vascular lock effect’ induce a hypoperfusion of the tumor area. The hypoxic microenvironment exacerbates oxidative stress in cancer cells by disrupting the balance of reactive oxygen species (ROS) production, ultimately leading to cellular damage [38,39].

Hypoxia-driven oxidative stress further amplifies the cytotoxic effects of ECT. The mechanism of action is primarily mediated through oxidative stress-induced DNA strand breaks, which stimulate ROS generation and lead to tumor necrosis [40,41]. Although ROS typically initiate apoptotic pathways via mitochondrial dysfunction, excessive ROS production triggered by BLM may overwhelm apoptotic mechanisms, leading to uncontrolled cell death [38,39]. While both BLM and OXP increase ROS levels, BLM-induced ROS generation is a primary mechanism of its cytotoxic action, directly causing DNA damage. In contrast, OXP induces ROS as a secondary effect, which contributes to its overall cytotoxicity and side effect profile [42].

Although prior studies have demonstrated the efficacy of OXP in treating CC531 tumors in rat models [43,44], the therapeutic potential of OXP in combination with rEP for CRLM has yet to be thoroughly investigated. In a study on melanoma cells, ECT enhanced the accumulation of platinum-DNA adducts by an average of 7.5-fold in vitro and 5.4-fold in vivo when OXP was used [45]. OXP enters tumor cells through multiple mechanisms, primarily via passive diffusion and, to a lesser extent, through active transport. Due to its neutral charge and lipophilic nature, OXP can traverse the lipid bilayer of the plasma membrane without the assistance of energy-dependent transporters [36]. This ability to passively diffuse allows for rapid initial uptake into cells, particularly in the early stages of exposure, where transporter-mediated mechanisms may not yet be upregulated [46,47]. The plasma membrane of CC531 rat colon carcinoma cells is composed of a phospho-lipid bilayer embedded with various proteins [48]. Consequently, we hypothesize that OXP exhibits increased permeability through the phospholipid tumor membrane, an effect that is not significantly enhanced by rEP.

The addition of BVZ led to a modest improvement in the antitumor efficacy of OXP, although outcomes remained statistically lower than those achieved with BLM. BVZ functions by binding to vascular endothelial growth factor A (VEGF-A), thereby blocking its interaction with endothelial cell receptors. This mechanism inhibits angiogenesis, restricting tumor growth and metastatic potential. Moreover, BVZ promotes vascular normalization, enhancing OXP delivery to the tumor site. By reducing vascular permeability and improving distribution, BVZ contributes to more effective drug penetration [49]. As a result, OXP demonstrated in our study superior oncological outcomes, including necrosis, proliferation, and microvessel density, when combined with BVZ compared to monotherapy.

Photoacoustic imaging enabled high-resolution 3D tomographic mapping of oxygen saturation by quantifying oxygenated and deoxygenated hemoglobin. The BLM-treated group exhibited significantly reduced SO_2_ and HbT levels compared to the other groups, reflecting the extensive tumor necrosis and the vascular-disrupting effect of BLM. These imaging findings were corroborated by immunohistochemical analyses, which showed a marked decrease in CD31-positive microvessels in the BLM group.

The mechanism of cell death following ECT remains unclear, and studies suggest that the cytotoxic effects of ECT are likely to be cell-specific [50,51]. In our previous studies, BLM with ECT caused extensive necrosis and to a limited extent also apoptosis [10,11]. On the contrary, OXP primarily induces apoptosis rather than necrosis in cancer cells. Its cytotoxicity is mainly driven by the formation of DNA-platinum adducts, which disrupt DNA replication and transcription, triggering intrinsic (mitochondrial) and extrinsic apoptotic pathways through activation of caspase-3, -8, and -9 [42]. Necrosis is less commonly associated with OXP and typically occurs only at high concentrations or under severe cellular stress, such as adenosine triphosphate depletion [52].

Regarding tumor proliferation, OXP represents partial retention of proliferative capacity, as indicated by moderate levels of PCNA expression [53]. In contrast, BLM exerts its antitumor effect primarily through DNA strand breaks caused by oxidative damage, leading to robust activation of DNA damage response pathways and more pronounced inhibition of cell proliferation.

This study offers valuable insights into the efficacy of ECT in CRLM. However, several limitations should be acknowledged. First, the experiments were conducted in an animal model. Thus, interspecies differences may limit the applicability of the findings to human biology, underscoring the need for validation in clinical trials. Second, the use of OXP monotherapy presents concerns regarding drug resistance and toxicity at higher doses, emphasizing the importance of exploring combination regimens such as FOLFOX or FOLFIRI. Third, the small sample size, aligned with the 3R principle, may have reduced the statistical power and generalizability of the results. Larger cohorts are necessary to better assess long-term efficacy and potential adverse events. Furthermore, the findings are specific to CRLM and may not extend to other hepatic tumors with differing vascular characteristics. Lastly, the short lifespan of rats limits the ability to evaluate long-term outcomes, such as tumor recurrence and late-onset toxicity.

## 5. Conclusions

Overall, our findings highlight the superior efficacy of BLM compared to OXP. While OXP remains a standard CRLM treatment, it was less effective than BLM, even with BVZ addition. These results underscore potential of BLM as a potent agent in ECT for the treatment of CRLM.

## Figures and Tables

**Table 1:** Blood sample analysis on day 13.

	BLM	OXP	OXP/BVZ
**Leukocytes (10^9^/L)**	9.5 ± 1.0	9.7 ± 0.7	8.9 ± 0.5
**Lymphocytes (10^9^/L)**	4.6 ± 0.3	4.6 ± 0.3	4.2 ± 0.2
**Monocytes (10^9^/L)**	0.9 ± 0.2	0.9 ± 0.2	0.8 ± 0.1
**Neutrophils (10^9^/L)**	4.1 ± 0.9	4.5 ± 0.6	4.1 ± 0.5
**RBC (10^12^/L)**	6.8 ± 0.2	6.6 ± 0.2	6.6 ± 0.2
**Platelets (10^9^/L)**	604.0 ± 36.0	584.0 ± 49.4	592.0 ± 38.2
**Hemoglobin (g/dL)**	13.1 ± 0.3	12.9 ± 0.2	13.3 ± 0.2
**Hematocrit (%)**	37.3 ± 1.0	36.4 ± 1.2	38.1 ± 0.8

Data are given as mean ± SEM.

## Data Availability

The data that support the findings of this study are available from the Institute for Clinical and Experimental Surgery in Saarland University (https://www.uniklinikum-saarland.de/de/einrichtungen/kliniken_institute/chirurgie/experimentalchirurgie (accessed on 15 July 2025). Further information is available from the corresponding author upon request.

## Abbreviations

The following abbreviations are used in this manuscript:

ECT	Electrochemotherapy
rEP	reversible Electroporation
BLM	Bleomycin
i.v.	Intravenous
CRLM	Colorectal Liver Metastases
OXP	Oxaliplatin
BVZ	Bevacizumab
SO_2_	Oxygen Saturation
HbT	Hemoglobin Concentration
PCNA	Proliferating Cell Nuclear Antigen
MPO	Myeloperoxidase
HPF	High-Power Field
SEM	Standard Error of the Mean
ANOVA	Analysis of Variance
RBC	Red Blood Cell
ROS	Reactive Oxygen Species
VEGF-A	Vascular Endothelial Growth Factor A
